# The development of a patient decision aid to reduce decisional conflict about antidepressant use in pregnancy

**DOI:** 10.1186/s12911-022-01870-1

**Published:** 2022-05-13

**Authors:** Neesha Hussain-Shamsy, Sarah Somerton, Donna E. Stewart, Sophie Grigoriadis, Kelly Metcalfe, Tim F. Oberlander, Carrie Schram, Valerie H. Taylor, Cindy-Lee Dennis, Simone N. Vigod

**Affiliations:** 1 Institute of Health Policy, Management and Evaluation, University of Toronto, 155 College Street, 4th Floor, Toronto, ON Canada; 2 Garry Hurvitz Centre for Brain and Mental Health, Hospital for Sick Children, 555 University Avenue, Toronto, ON Canada; 3 Centre for Mental Health, University Health Network, Toronto General Hospital Research Institute, 200 Elizabeth Street, Toronto, ON Canada; 4 Temerty Faculty of Medicine, University of Toronto, 1 King’s College Circle, Toronto, ON Canada; 5 Sunnybrook Health Sciences Centre and Research Institute, 2075 Bayview Avenue, Toronto, ON Canada; 6 Women’s College Hospital and Research Institute, 76 Grenville Street, Toronto, ON M5S 1B2 Canada; 7 Lawrence S. Bloomberg Faculty of Nursing, University of Toronto, 155 College Street, Toronto, ON Canada; 8 BC Children’s Hospital, 4500 Oak Street, Vancouver, BC Canada; 9 Department of Pediatrics, Faculty of Medicine, University of British Columbia, 317-2194 Health Sciences Mall, Vancouver, BC Canada; 10 Department of Psychiatry, University of Calgary, 2500 University Dr NW, Calgary, AB Canada

**Keywords:** Patient decision aid, Pregnancy, Antidepressant, Depression, Online

## Abstract

**Background:**

People with moderate to severe depression in pregnancy must weigh potential risks of untreated or incompletely treated depression against the small, but uncertain risks of fetal antidepressant drug exposure. Clinical support alone appears insufficient for helping individuals with this complex decision. A patient decision aid (PDA) has the potential to be a useful tool for this population. The objective of our work was to use internationally recognized guidelines from the International Patient Decision Aids Standards Collaboration to develop an evidence-based PDA for antidepressant use in pregnancy.

**Methods:**

A three-phased development process was used whereby, informed by patient and physician perspectives and evidence synthesis, a steering committee commissioned a web-based PDA for those deciding whether or not to start or continue antidepressant treatment for depression in pregnancy (Phase 1). A prototype was developed (Phase 2) and iteratively revised based on feedback during field testing based on a user-centred process (Phase 3).

**Results:**

We developed a web-based PDA for people deciding whether to start or continue antidepressant use for depression in pregnancy. It has five interactive sections: (1) information on depression and treatment; (2) reasons to start/continue an antidepressant and to start/stop antidepressant medication; (3) user assessment of values regarding each issue; (4) opportunity to reflect on factors that contribute to decision making; and (5) a printable PDF that summarizes the user’s journey through the PDA.

**Conclusions:**

This tool, which exclusively focuses on depression treatment with Selective Serotonin Reuptake Inhibitors and Serotonin–Norepinephrine Reuptake Inhibitors, can be used by individuals making decisions about antidepressant use to treat depression during pregnancy. Limitations of the PDA are that it is not for other conditions, nor other medications that can be used for depression, and in its pilot form cannot be used by women who do not speak English or who have a visual impairment. Pending further study, it has the potential to enhance quality of care and patient experience.

**Supplementary Information:**

The online version contains supplementary material available at 10.1186/s12911-022-01870-1.

## Introduction

Depression is a common complication of pregnancy, affecting up to 10% of pregnancies in Canada [1]. Untreated or incompletely managed, depression in pregnancy can have a serious impact on both child and maternal health, including associations with childhood health and developmental problems that extend past the neonatal phase, increased risk of chronic maternal depression (which in turn has other downstream impacts on maternal and child health), and, in rare cases of severe depression, suicide [2–6]. Women with depression in pregnancy are also at high risk for postpartum depression which increases the likelihood that their children may have higher rates of poor developmental and emotional outcomes as a result of depression-associated impaired maternal-infant interactions [7–12]. Due to the negative consequences of depression in pregnancy, there is urgency to ensure effective treatment. This often requires the mother to make complex decisions about treatment in an effort to balance her own mental health with the health and well-being of her unborn child. Despite depression being one of the most common morbidities in pregnancy, there is evidence that as few as 12% of women receive any type of treatment [13].

The standard types of treatment for depression in pregnancy are psychotherapy and antidepressant medication. Psychotherapy is indicated for mild and moderate depression but can take several weeks or months to have an effect. In the interim, this leaves the mother and fetus vulnerable to the effects of untreated depression and alone, it is unlikely to result in substantive improvements if the depression is moderate or severe [14]. First-line antidepressant medications (Selective Serotonin Reuptake Inhibitors, SSRIs, or Serotonin-Norepinephrine Reuptake Inhibitors, SNRIs) are effective for treatment of depression and prevention of relapse, with approximately 67% of individuals achieving remission with antidepressant use [14–19]. As many as 68% of those who stop antidepressant use in pregnancy relapse, exposing themselves and their infants to the effects of untreated depression [20]. Unfortunately, exposure to these drugs has been associated with small, increased risks of adverse neonatal outcomes [21–30]. Spontaneous abortions (i.e. miscarriage), low birth weight, and preterm birth have all been reported in exposed infants [31], but these risks may or may not be higher than among mothers with untreated depression [4]. It is difficult to separate effects of genetics and maternal mood from long term effects of in-utero exposure to SSRIs and SNRIs given that these drugs cross the placenta, the fetal blood–brain-barrier, and cause changes in fetal motor and sleep behaviours, suggesting long-term impact is possible [32, 33]. For pregnant individuals who are unlikely to respond to psychotherapy alone, the potential risks of un- or incompletely treated depression must be weighed against the small, but still somewhat uncertain risks of fetal antidepressant drug exposure.

In keeping with a patient-centred approach to medical care, clinicians must consider the personal needs and values of the pregnant person when deciding on a treatment approach; in turn, they also *want* to be involved with these decisions [34, 35]. Yet, even after telephone counseling from teratology information services, or psychiatric clinical care, high levels of “decisional conflict”, a construct associated with emotional distress as well as delayed, and sometimes ineffective treatment decisions, remain [36–38]. Patient-focused interventions such as patient decision aids (PDAs) to assist those who are deciding whether or not to take antidepressants in pregnancy are urgently needed, given such negative consequences of untreated (or under-treated) depression.

PDAs are interventions that are designed to facilitate effective decision-making and reduce decisional conflict for patients who are facing complex health-related decisions [39]. They assist in understanding available options, possible benefits and harms, and allow consideration of options from a personal view (e.g. how important the possible benefits and harms are to them) in order to be better prepared to participate in decision-making with their provider [40]. Systematic reviews have found that patients using PDAs are more knowledgeable about their options, have more accurate expectations of possible benefits and harms, make decisions more consistent with their informed values, and participate more actively in the decision-making process [41]. To our knowledge, no previous PDA for antidepressant use in pregnancy had been developed using guidelines from the International Patient Decision Aids Standards (IPDAS) Collaboration and has undergone rigorous evaluation to establish its efficacy at reducing related decisional conflict. These guidelines provide a framework for developing high-quality, evidence-based PDAs as well as standards for determining whether or not the PDA was subject to a rigorous development and evaluation process. The aim of this paper is to outline the systematic development of a patient decision aid for antidepressant use in pregnancy framed on the International Patient Decision Aids Standards guidelines.

## Methods

**Fig. 1:**
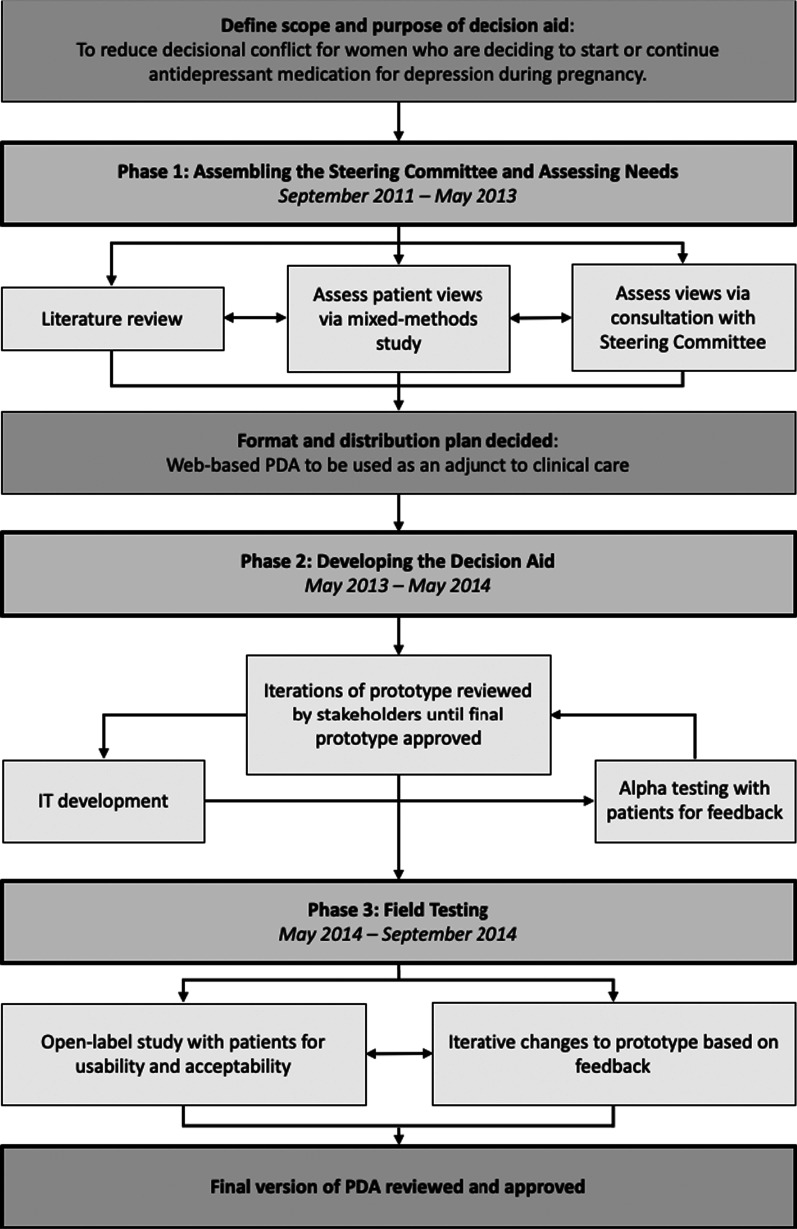
The development process used for this PDA on antidepressant use in pregnancy, adapted from the IPDAS model development process for decision aids

The IPDAS Guidelines have a model development process for decision aids [42] that can be divided into three phases: (1) assembling the steering group and assessing needs, (2) drafting the decision aid, and (3) field testing. These sections are prefaced by formally defining the scope and purpose of the decision aid and identifying the target audience (Fig. 1).

### Scope, purpose and audience

The overall purpose of the PDA is to reduce decisional conflict for individuals who are determining if they should start or continue their antidepressant medication during pregnancy. It is designed to be an adjunct to clinical care in multiple contexts, including by primary care providers and general psychiatrists in the pre-pregnancy planning stage, as well as antenatal care providers who prescribe during pregnancy itself [43]. The anticipated users are those who are pregnant or planning a pregnancy, deciding about antidepressant (SSRI or SNRI) use to treat depression, and having difficulty with their decision, although it may also be helpful for those who are not necessarily experiencing decision-making difficulty but simply wish for more information or assistance with clarifying their treatment preferences.

### Phase 1: Assembling the steering group and assessing needs

A steering committee including international representation from experts in the field was assembled. Members included those from the perinatal mental health, psychopharmacology, family medicine, and obstetrics and gynecology fields, as well as community partners and end users. Based on the IPDAS guidelines, a four-step design process was followed. First, a literature review to synthesize evidence on depression, antidepressants, and decision-making in pregnancy was conducted. Second, a mixed-methods research study was commissioned to determine patient perspectives on decisional needs related to antidepressant use in pregnancy. The study found that elevated levels of decisional conflict about antidepressant use in pregnancy remained, even after consultation with a perinatal psychiatrist [37]. It also identified barriers, including difficulty weighing maternal versus infant health, lack of high-quality information, negative external influences, and emotional response to decision-making, as well as facilitators, including interpersonal support, availability of allied mental health support, and severe depressive symptoms, to decision-making [37]. Third, members of the steering committee provided their input in May 2013 on their views of needs in light of the evidence gathered and reviewed an interactive prototype developed by the study team. Finally, informed by the IPDAS guidelines, and relevant evidence on depression and antidepressant use in pregnancy, decisional needs, and decision-making during pregnancy, the steering committee determined to move forward with the development of an interactive, web-based PDA that would function as an adjunct to clinical care.

### Phase 2: Drafting the decision aid

Informed by evidence gathered in Phase 1, the study team continued development of a working prototype for the PDA by drafting the PDA content and consulting with the IT vendor on design and layout. Initial decisions related to the text content, design, and usability were informed by IPDAS consensus-driven research on information presentation, the subject matter expertise of our IT vendor and the research team [44–47]. Alpha testing for initial feedback with patients were conducted. Best practice guidelines for numerical literacy and risk communication were followed (e.g. using absolute risks, and consistent proportion denominators) [48, 49]. Participant stakeholder feedback was sought and determined that sliding Likert Scales were preferred to visualize personal elements of the decision-making process (e.g. how far they were leaning in terms of their decision to use an antidepressant versus to not use an antidepressant, and for how much different risks and benefits mattered to them), whereas numerical representations were preferred for risks and benefits related to safety and efficacy due to the volume of information. In order to give participants control over how much information could be seen at once, basic information was presented on a page with the option to “hover over” for additional details. The written content of the PDA was designed at a Grade 6 reading level. This process took approximately one year (May 2013-May 2014) until a workable design was developed. The online design and content were iteratively reviewed and edited by stakeholders (including 2–3 patient stakeholders) until they were satisfied that the PDA was ready for field testing (i.e. beta testing).

### Phase 3: Field testing

**Fig. 2:**
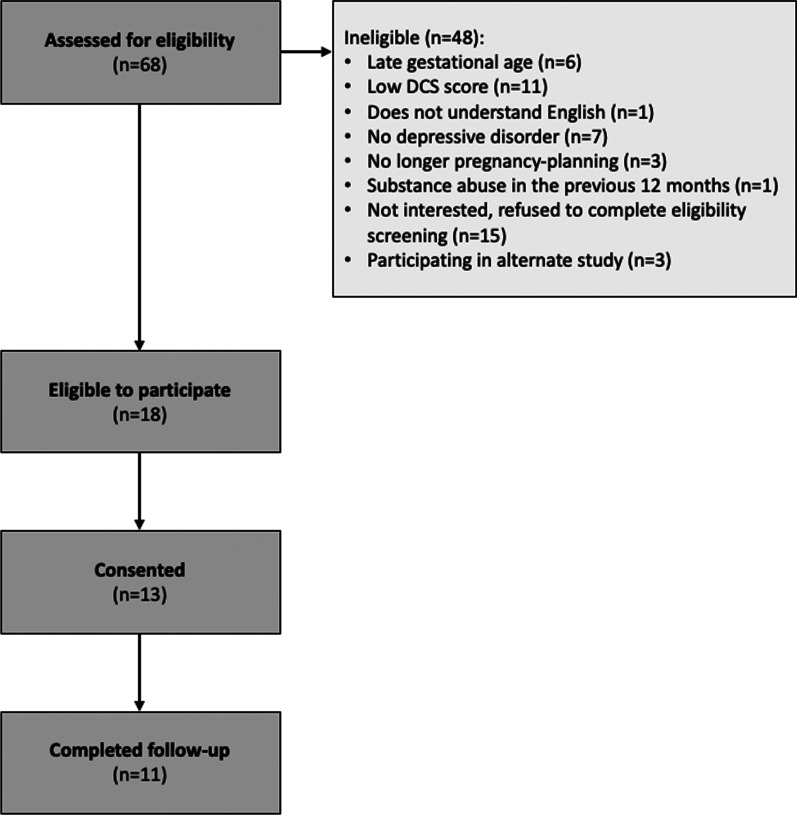
Participant flow through field testing (Phase 3)

The PDA was field tested in an open-label study over five months at Women’s College Hospital in Toronto. The goal of this study was to determine the usability and acceptability of the PDA, make necessary modifications to the tool, and inform the methodology of larger scale evaluations. Adults were recruited if they were: (1) pregnant or planning a pregnancy (< 30 weeks gestation at enrollment), (2) had been offered to either start or continue an SSRI or SNRI as treatment for depression by their clinical provider, and (3) experiencing high decisional conflict (determined by their score on the decisional conflict scale (DCS) at eligibility screening). The DCS is a validated tool for measuring uncertainty with making a health-related decision [50]. Each of the 16 items in the DCS is scored from 0 (strongly agree) to 4 (strongly disagree). These are summed, divided by 16 and multiplied by 25 to give an overall DCS score ranging from 0 (no decisional conflict) to 100 (extremely high decisional conflict); a score of > 37.5 is associated with ineffective decision-making [51]. Potential participants were excluded from the field testing study if they: (1) had alcohol or substance abuse or dependence in the past 12 months, or (2) had active suicidal ideation or psychosis, or (3) had any major obstetrical complications or fetal cardiac anomalies in the current or in a past pregnancy (as this changes the risk/benefit ratio in regards to antidepressant use), or (4) were visually impaired, or (5) were unable to read, speak, or understand English. After providing informed consent, participants completed a baseline questionnaire, were given access to the PDA website, and completed a follow-up questionnaire four weeks later. Qualitative data were rapidly and iteratively sought and analysed in order to address common feedback which could then be field tested by subsequent participants. Participants were recruited until data saturation was reached and no new or substantial feedback was being received. Overall, 68 people were referred to the study for eligibility screening. There were 48 women who were ineligible for the study due to late gestational age (n = 6), low DCS score (n = 11), no English language ability (n = 1), no depressive disorder (n = 7), refusal to complete eligibility screening (n = 15), substance abuse (n = 1), or no longer pregnancy-planning (n = 3). An additional three were referred to participate in another study and could not do both. Of the eighteen individuals who were eligible to participate in the study, thirteen people agreed to provide their informed consent and eleven completed follow-up (Fig. 2). The majority of participants agreed the PDA was helpful for decision-making about antidepressant use in pregnancy and would recommend it to others. Exercising a user-centred approach, changes were made on an ongoing basis; most feedback related to clarifications in wording and formatting for ease of use. For example, one participant noted that they “…didn’t like the decision meter on every page.” As a result, it was removed from pages where it was felt to be redundant or unnecessary, such as on the page where users can clarify factors that contribute to their decision making (Additional file 1: Appendix 1). In general, feedback was very positive and noted the importance of such a tool to support decision making. For example, one participant in field testing said, “I feel a lot better about my ability to justify my decision to others.” The final version was reviewed and approved by the study team.

### Funding and ethics

Funding for the various stages of this process was provided by the Alternative Funding Plan Innovation Fund at Women’s College Hospital and the Canadian Institutes of Health Research. All research studies conducted as part of this process were approved by the Research Ethics Board at Women’s College Hospital.

## Results

Using the systematic development process outlined by the IPDAS guidelines, we developed an interactive, web-based PDA for those deciding whether to start or continue antidepressant use for depression in the context of pregnancy. This PDA takes approximately 20–30 min to view online in its entirety. It is designed to be viewed on a computer.

Access to the PDA is via a unique log-in comprised of a username and password. During field testing this was assigned to users by the research assistant. In the first few pages of the PDA, the user is asked to select whether or not they are choosing to start an antidepressant or to continue an antidepressant. This selection sets the context for how information is laid out throughout the remainder of the PDA.

The PDA is divided into five sections. The first section of the PDA offers information on depression, including a basic overview, the epidemiology of depression, and information on when starting or continuing an antidepressant might be recommended. The next section outlines reasons to start (or continue) an antidepressant and reasons not to start (or to stop) antidepressant medication. This information is provided to patients in table form, where each reason in the table is listed in point form, with an option to hover over key words in order to read more information about that issue. The PDA was designed in this manner based on conflicting initial feedback during alpha testing where some users wanted to know more information and detail about the issue, and others preferred to know basic information only. In this manner, patients are able to choose for themselves the level of information that they are comfortable with for their own decision making.

The third section allows the user to assess their own values in regard to each issue. Value clarification exercises allow users in this context to explore which of the risks and benefits related to antidepressant use in pregnancy matter most to them [52]. Here, the same issues as in Sect. 2 are laid out in the same format, alongside sliding scales that allow each issue to be ranked from “Does not matter” to “Matters a lot”. At the bottom of each of these pages is a heading that says, “After reading about your options, where are you in your decision making process?” Beneath this heading is a slider that allows the user to gauge where they currently are in their decision making process from “I will definitely not take antidepressant medication” to “I will definitely start taking antidepressant medication”. The selection on the bottom of each page carries over to the next page, allowing users to adjust how they feel on an ongoing basis, based on the new information they read. Each page also has links to references as appropriate.

The next section of the PDA gives users the opportunity to reflect on factors that contribute to their decision making. Understanding who or what is making a decision harder or easier to make can help facilitate decision making. For example, if an influence is helpful, it can be called on for support, whereas if it is not helpful, this can be explicitly identified in order to help the patient make a more independent decision. Five potential influences (friends and family, partners, media, culture, and providers) are listed along with a tool to select if that particular influence it is helpful, not helpful, or if it does not affect them. The selection is made by clicking on a green smiling face, a red sad face or a grey neutral face. Clicking the name of each potential influence on the left hand side of the screen brings up a description of, and anonymized quotes from others about that influence on the right hand side of the screen, taken from the decisional needs study conducted in Phase 1 [37]. Users are also able to add custom influences beyond the five provided.

The final section is a printable PDF that summarizes the user’s journey through the PDA, including how they have ranked each of the reasons for starting and not starting (or continuing and stopping) antidepressant medication, and their perceived influences. This sheet can be downloaded and saved, or printed and discussed with the patient’s clinician in order to have a more informed discussion about how the individual values their treatment options.

## Discussion

The systematic development of an evidence-based PDA for antidepressant use is pregnancy is a feasible endeavour. To our knowledge, ours is the first interactive PDA for antidepressant use in pregnancy that has been developed according to the IPDAS framework and has undergone rigorous evaluation. The initial results of field testing support further testing of the PDA as an adjunct to clinical care for pregnancy-planning and pregnant people having difficulty making decisions about antidepressant use in pregnancy. These findings were echoed in the results of a pilot randomized controlled trial assessing the feasibility of a trial protocol to definitively evaluate the effectiveness of this PDA in a Canadian context [43] as well as in the United Kingdom [53]. A large-scale national (Canadian) evaluation of this tool to determine its efficacy in facilitating decision-making is currently underway (ClinicalTrials.Gov: NCT03632863).

There are several potential implications of this tool. Our PDA has the potential to fill an important gap in decisional needs related to the treatment of depression in pregnancy. Our work is rooted in the principle of decisional conflict, high levels of which are associated with distress and delayed decision-making. This has been found in several perinatal studies on antidepressant use [34, 37]. The literature also affirms that there is an urgent need to develop interventions for those faced with this decision. Additionally, this tool can easily be adapted for use in other countries (i.e. trade names for medications), and scaled to reach the large number of women who are having clinical consultations about antidepressant use in pregnancy across Canada with minimal training needed for clinicians who might recommend using this tool to their patients. Finally, this tool has the potential to change the clinical management of individuals considering antidepressant use in the context of pregnancy as it may provide information and exercises to help people clarify their own values about their treatment options, in order to make a more effective and timely decision about the management of their mental health in pregnancy.

Evidence shows user-centred design approaches lead to higher-quality interventions, increased user acceptance, and improved efficiencies due to the early identification and rectification of usability problems before the launch of the intervention [54]. IPDAS recommends the publication of the development (including design and field testing) process [55], of patient decision aids developed using its framework, in addition to publishing results of evaluation efforts; for this PDA, pilot feasibility results are published elsewhere [35] and an efficacy evaluation is currently underway (ClinicalTrials.Gov: NCT03632863). Our study provides an example of a systematic, yet user-centred approach to the design and development of a virtual health intervention, which is an important consideration given the increased use of virtual health interventions as a result of the COVID-19 pandemic and their likely integration into mainstream healthcare delivery practices as the pandemic subsides [54, 56].

There are some limitations to our PDA. While SSRI and SNRI antidepressants are commonly prescribed for other mental health conditions, such as anxiety, the information provided in our PDA focuses on depression exclusively. In addition, some may be prescribed other types of antidepressants to treat depression, including tricyclic antidepressants, which are also not discussed in this PDA. Those who do not speak English or who have a visual impairment that precludes their ability to read and understand information on an online website will not be able to use the PDA in its current form. As well, it has not been optimized for mobile use which may limit accessibility or experience for those who do not have access to a laptop or desktop computer. Finally, we are unable to report on the diversity of the sample used in field testing, however the sample studied in the pilot randomized controlled trial was diverse with respect to languages spoken, countries of origin, and cultural diversity (43). The strengths of our PDA are its foundation in evidence-based research on depression and antidepressant use in pregnancy, as well as its capability to be updated as our scope of knowledge on the impact of antidepressant use and depression in pregnancy evolves. It can be widely accessible given its online nature, and can additionally be accessed with discretion for those that may be faced with the stigma of having a mental health condition in pregnancy, or with another individual if they so choose (such as a partner who may not be present during a clinical consultation).

## Conclusions

In conclusion, this tool is an easily scalable intervention that can be used as an adjunct to clinical care for individuals who are making decisions about antidepressant use to treat depression during their pregnancy, thereby enhancing quality of care and patient experience. Rigorous evaluation of the tool is underway.

## Supplementary Information

**Additional file 1.** Sample screenshots of the PDA prior to and after field testing was completed and patient feedback incorporated.

## Data Availability

This manuscript describes the development process for a patient decision aid. Participant permission to deposit data in a public data repository was not obtained at the time data was collected. The datasets used and analysed during the current study are available from the corresponding author on reasonable request.

## Abbreviations

SSRI: Selection serotonin reuptake inhibitor
SNRI: Serotonin–norepinephrine reuptake inhibitor
PDA: Patient decision aid
IPDAS: International Patient Decision Aids Standards
DCS: Decisional conflict scale
