# Supplementary material for: The development of a patient decision aid to reduce decisional conflict about antidepressant use in pregnancy
Source: BMC Med Inform Decis Mak. 2022 May 13;22:130. doi: 10.1186/s12911-022-01870-1 (PMC9099318; doi:10.1186/s12911-022-01870-1)
Supplement: Supplementary file 1 — Additional file 1. Sample screenshots of the PDA prior to and after field testing was completed and patient feedback incorporated. [file 12911_2022_1870_MOESM1_ESM.pdf]

Appendices

Appendix 1: Sample screenshots of the PDA prior to and after field testing was completed and patient feedback incorporated. (Note that some content has been blurred to preserve the integrity of ongoing evaluations).

Appendix Figure 1a

**Before field testing**		
Each risk was presented on a separate page in the context of having "untreated depression" versus when using antidepressant medication, with colourful, interactive sliders for the patient to note how much of a worry that risk is to them. In this example, the risks of prematurity and low birth weight are presented.	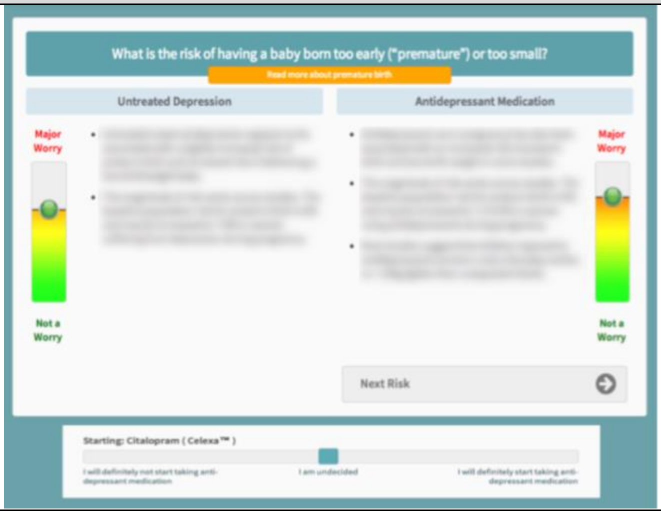	
**Sample feedback**	• "Wanted more info on risks and benefits of both options."<br>• "Sometimes having two columns of information side by side was overwhelming, would be better to separate it on different pages."	
**After field testing**		
Patients noted that the language around "untreated depression" versus using antidepressant medication was confusing. The language was changed throughout to reflect the decision being made (i.e. either "starting antidepressant medication" versus "not starting antidepressant medication" OR "continuing antidepressant medication" versus "stopping antidepressant medication"). Patients preferred to see all risks on one page, and to read more information about each risk by hovering their cursor over it. Feedback received by users also suggested that it would be more helpful to have a separate page in which the patient could use sliders to note how much each risk mattered to them (versus how much of a worry it was), with all risks presented together. Links to references were added.	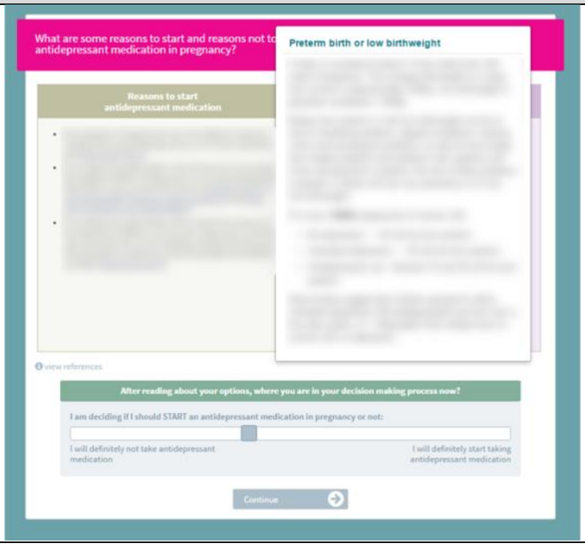	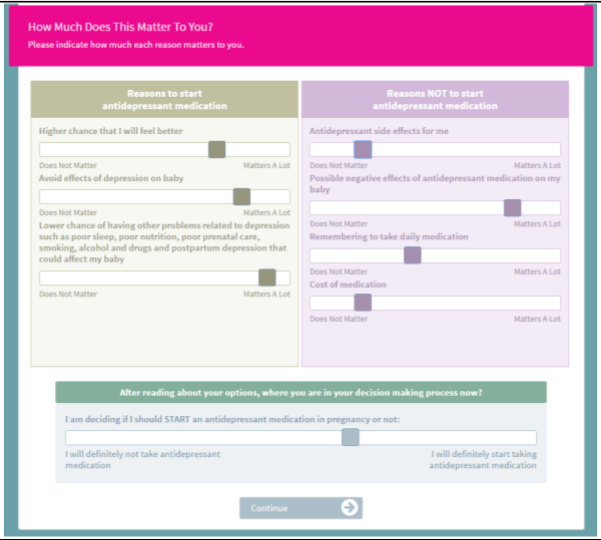

Appendix Figure 1b

**Before field testing**	
In this example, the role of external influences on decision making are presented. A sample of influences on decision making are presented in a list, alongside quotes from the mixed-methods study on patient perspectives. Users are invited to colour code each influence based on how each influence impacts their decision making by making them lean towards or away from a treatment option. Space is provided for other influences which may not be captured in the list provided.	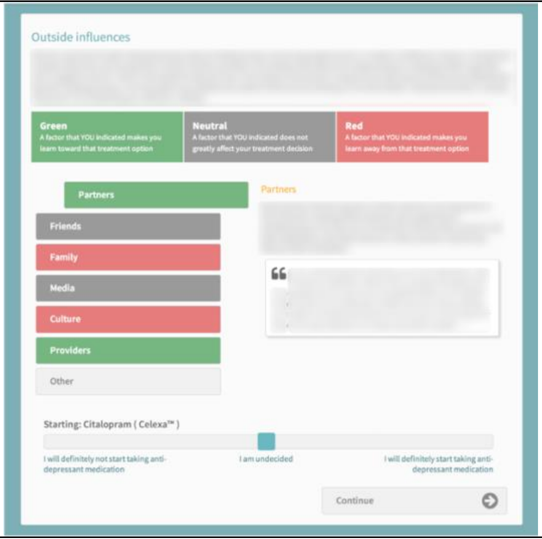
**Sample feedback**	• "The contrast between colours was hard to look at."<br>• "Didn't like the decision meter on every page."
**After field testing**	
The wording in the introductory text was simplified based on user feedback. Happy, sad, and neutral faces were substituted for colour each influence to increase accessibility and add visual interest. Language was simplified and modified to look at each external influence as helpful versus not helpful in the decision making process. Additional quotes, where available, were added. Links to references were added.	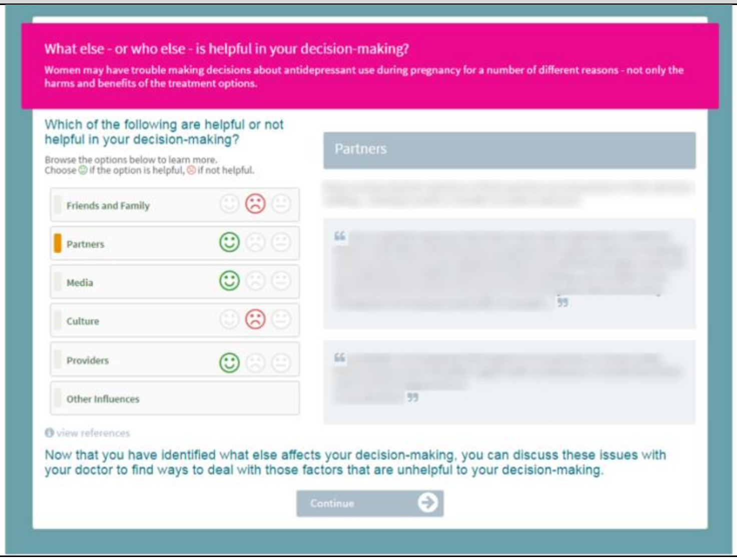
